# Impact of baseline body composition on prognostic outcomes in urological malignancies treated with immunotherapy: a pooled analysis of 10 retrospective studies

**DOI:** 10.1186/s12885-024-12579-x

**Published:** 2024-07-11

**Authors:** Wangbin Ma, Qiao Shi, Lilong Zhang, Zhendong Qiu, Tianrui Kuang, Kailiang Zhao, Weixing Wang

**Affiliations:** 1 Department of General Surgery, Renmin Hospital of Wuhan University, Wuhan, China; 2 Hubei Key Laboratory of Digestive System Disease, Wuhan, China; 3 Laboratory of General Surgery, Renmin Hospital of Wuhan University, Wuhan, China

**Keywords:** Immune checkpoint inhibitors, Body composition, Skeletal muscle index, Psoas muscle index, Sarcopenia, Urological malignancies

## Abstract

**Objective:**

Numerous epidemiological investigations have explored the impact of body composition on the effectiveness of immune checkpoint inhibitors (ICIs) in urological malignancies (UM) patients, yielding conflicting findings. As a result, our study aims to elucidate the influence of baseline body composition on the long-term prognosis of UM patients treated with ICIs.

**Methods:**

We employed a rigorous systematic search across various databases, including PubMed, Embase, the Cochrane Library, and Google Scholar, to identify studies meeting our inclusion criteria. Our primary endpoints of interest encompassed overall survival (OS) and progression-free survival (PFS).

**Results:**

This analysis included a total of 10 articles with a combined patient cohort of 707 individuals. Our findings revealed a noteworthy association between several body composition parameters and unfavorable OS outcomes, including low psoas muscle index (PMI; HR: 3.88, *p* < 0.001), low skeletal muscle index (SMI; HR: 1.63, *p* < 0.001), sarcopenia (HR: 1.88, *p* < 0.001), low visceral adipose index (VAI; HR: 1.38, *p* = 0.018) and low subcutaneous adipose index (SAI; HR: 1.37, *p* = 0.018). Furthermore, our analysis demonstrated that low PMI (HR: 2.05, *p* = 0.006), low SMI (HR: 1.89, *p* = 0.002), sarcopenia (HR: 1.80, *p* < 0.001), and low VAI (HR:1.59, *p* = 0.005) were significantly correlated with inferior PFS. Conversely, SAI did not manifest a pronounced association with PFS in UM patients treated with ICIs.

**Conclusion:**

Collectively, our study findings underscore a substantial relationship between baseline body composition and reduced clinical efficacy in UM patients undergoing ICI therapy.

**Supplementary Information:**

The online version contains supplementary material available at 10.1186/s12885-024-12579-x.

## Introduction

Urological malignancies (UM), which include renal cell carcinoma (RCC), bladder cancer (BC), prostate cancer (PC), and urothelial cancer (UC), are a significant global public health issue [1]. Various treatments and techniques have made progress in managing UM, their clinical prognosis has significantly improved over the last two decades [2]. However, the development of ICIs has revolutionized the treatment of many cancers, including UM.

ICIs have become a widely adopted treatment modality for various types of cancer, especially for recurrent and metastatic diseases that are resistant to conventional therapies. The indications for ICIs have been expanding in recent years [3]. In particular, having been approved for the treatment of RCC and UC, ICIs have shown significant improvements in patient survival compared to traditional therapies [4, 5]. ICIs can also provide long-lasting disease control and extend survival, even for patients with advanced disease and disease progression. Companion and complementary diagnostics have been developed to aid in the identification of patients who are most likely to benefit from ICIs [6]. All immune checkpoint inhibitors, except ipilimumab, block PD-1/PD-L1 interaction [7]. While PD-L1 assessment through immunohistochemistry is used for companion diagnostics, its predictive accuracy is limited due to heterogeneous expression in tumors [7]. Other biomarkers like tumor mutation burden and microsatellite instability have limited predictive value when used alone [6], prompting researchers to investigate additional biomarkers [8].

Skeletal muscle wasting is a hallmark of cancer cachexia and sarcopenia and has been correlated with treatment outcomes in cancer patients [9–12]. Furthermore, the depletion of skeletal muscle mass in patients undergoing neoadjuvant therapy has been recognized as an adverse prognostic indicator for the overall survival of individuals with ovarian, esophageal, and foregut cancers [13–15]. Whereas, it is unclear whether skeletal muscle loss during ICI treatment affects the prognosis of UM patients. Although abdominal adipose tissue has been associated with cancer prognosis, its relationship with ICI therapy outcomes in UM patients remains incompletely understood [16]. Therefore, it would be worthwhile to investigate the impact of changes in body composition during ICI treatment on the clinical outcomes of UM patients.

The aim of this investigation is to perform an extensive review and meta-analysis to explore the correlation between body composition and unfavorable outcomes in patients with UM. Our study is anticipated to constitute the inaugural comprehensive assessment of the connection between body composition and the prognosis of UM patients undergoing ICI treatment.

## Methods

### Literature search strategies

The current study adheres to the Preferred Reporting Items for Systematic Reviews and Meta-Analyses (PRISMA) guidelines [17]. A comprehensive literature search was performed in the PubMed, EMBASE, and Cochrane databases from January 2024 onwards. The following keywords were used: “immune checkpoint inhibitors” [Mesh], “skeletal muscle index”, “psoas muscle index”, “sarcopenia”, “subcutaneous adipose index”, “visceral adipose index”, and “intramuscular adipose index”. Supplementary Table 1 provides detailed information on the search strategies. In addition, Google Scholar was searched to locate unpublished research data. Finally, we manually searched the reference lists of eligible papers.

### Eligibility criteria

The inclusion criteria were: include UM patients treated with ICIs; evaluate the prognostic value of body composition parameters such as skeletal muscle index (SMI), psoas muscle index (PMI), sarcopenia, subcutaneous adipose index (SAI), visceral adipose index (VAI), and intramuscular adipose index (IAI); provide data on OS and PFS; be published as full-length articles in peer-reviewed journals. Studies that were only available as abstracts, comments, or case reports were excluded. If there were studies that reported on the same patient population, we included only the study that had the most comprehensive data and used rigorous methods in the meta-analysis.

### Data extraction

The data extraction mainly was focused on: the first author’s name, year of publication, country of origin, study design, sample size, male/female, age (years), type of cancer, treatment, diagnostic method, outcomes, and definitions. Preferential extraction of multivariate analysis data was done for HR analysis over univariate analysis.

### Methodological quality assessment

The quality of each study was evaluated using the Newcastle-Ottawa Scale (NOS) [18]. The domains of patient selection, study comparability, and study endpoints were assessed according to tailored quality criteria, and a maximum score of nine was possible. A score greater than 7 indicated high quality, while a score between 5 and 7 indicated moderate quality. Studies with a score less than 5 were considered low quality.

### Statistical methods

HRs and their corresponding 95% CIs were utilized for the amalgamation of data. To gauge statistical heterogeneity, the chi-squared test was employed. A random-effects model was adopted when the p-value was less than 0.1 and the I^2^ statistic exceeded 50%, denoting substantial heterogeneity; otherwise, a fixed-effects model was applied. A sensitivity analysis was conducted to assess the robustness of the results by systematically excluding individual studies. Publication bias was evaluated using Begg’s and Egger’s tests. A significance threshold of *p*-value < 0.05 was considered as indicative of statistical significance.

## Results

### Study retrieved and study characteristics

**Fig. 1:**
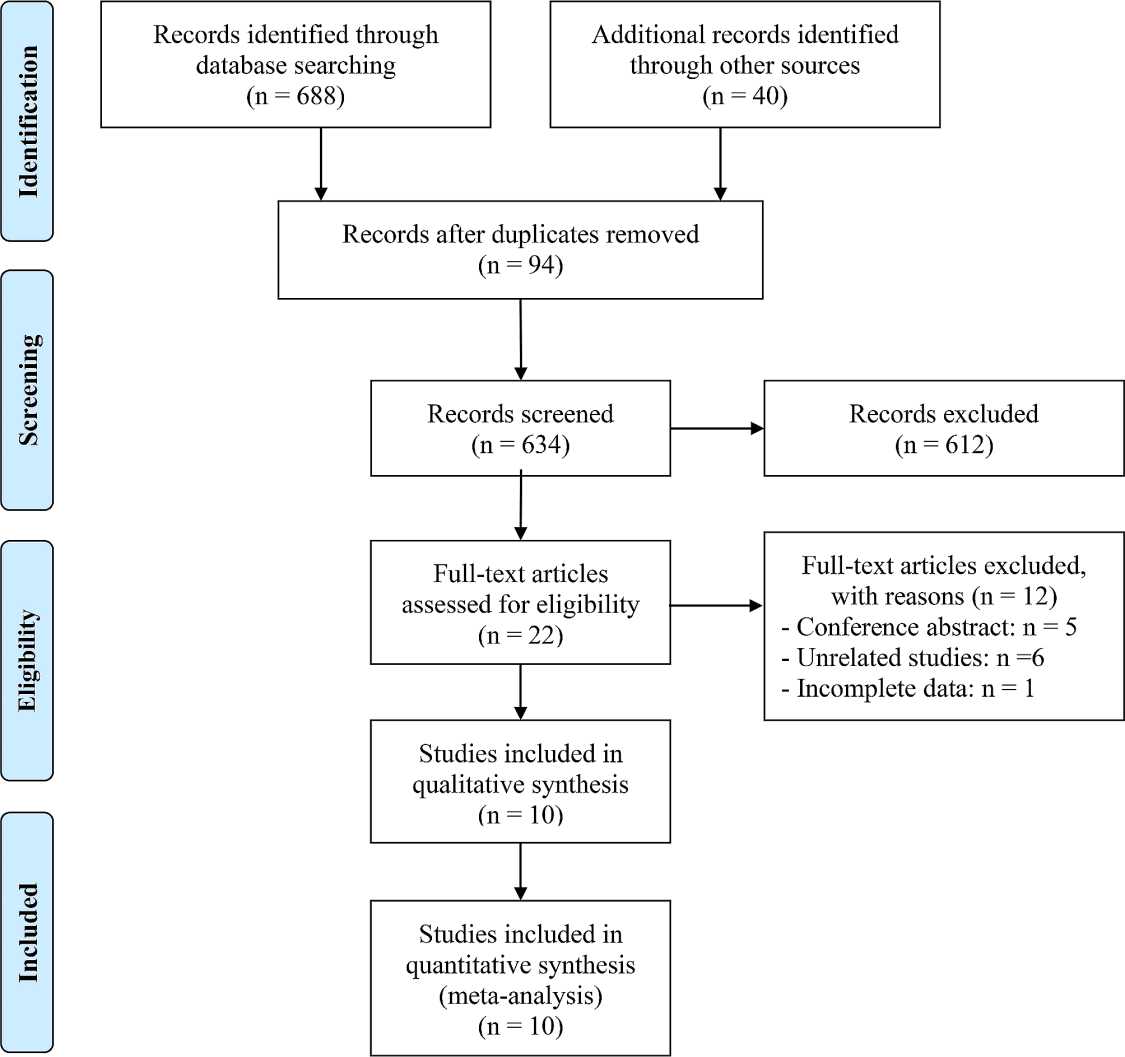
The flow diagram of identifying eligible studies

A total of 728 articles were initially collected through a systematic literature search. After screening 22 full-text articles, we included 10 retrospective studies [19–28] published after 2020, reporting a total of 707 μm patients who received ICIs. Table 1 provides the main characteristics of the included studies. All the included articles were exclusively in the English language. All studies included in this analysis were retrospective studies. Five studies were conducted in Japan [20, 23, 26–28] and three in the USA [21, 24, 25], while another two studies were conducted in Turkey [19] and France [22]. Six studies [19, 21–24] focused on RCC, while four studies [20, 25–27] examined UC (Table 1). Sarcopenia was measured using the SMI in seven studies [20–26, 28], while the PMI was used to define sarcopenia in two studies [23, 27] and both the SMI and PMI were used in one study [23] (Table 1). Among these studies, eight were of high quality (seven or eight scores), while two were of medium quality (score of six). The flow chart and reasons for exclusion are illustrated in Fig. 1.

**Table 1:** Main characteristics of the studies included

Study	Study design	Study region	Study period	Sample size	Age	Male/female	Site	Treatment line	Treatment	Follow-up (months)	Body Composition Variables	Outcomes
Aslan et al. 2022 [19]	R	Turkey	10/2010-10/2021	52	30/22^d^	38/14	RCC	II	Nivolumab	11.4 (0.7–63)^a^	SAI	OS, PFS
Fukata et al. 2022 [20]	R	Japan	02/2018-03/2021,	44	70 (54–80)^a^	30/14	UC	II	Pembrolizumab	13.2(1–40.8)^a^	SMI	PFS
Ged et al. 2022 [21]	R	USA	07/2011-04/2020	205	63 (40–90)^a^	152/53	RCC	I/II	ICIs	31.2 (1-77.8)^a^	SMI, VAI, SAI	OS
Herrmann et al. 2022 [22]	R	France	2016–2020	46	66 (37–86)^a^	31/13	RCC	II	Nivolumab	16^c^	SMI	OS
Ueki et al. 2022 [23]	R	Japan	12/2016-10/2020	96	65/31^e^	71/25	RCC	II	Nivolumab	9.7 (0.3–48.4)^a^	PMI, SMI	OS, PFS
Martini et al. 2021(RCC) [24]	R	USA	2015–2020	79	61.0^c^	58/21	RCC	I/II	ICIs	-	SMI, VAI, SAI, IAI	OS, PFS
Martini et al. 2021(UC) [25]	R	USA	2015–2020	70	69.5^c^	49/21	UC	I/II	ICIs	20.1^c^	SMI, VAI, SAI, IFI	OS, PFS
Fukushima et al. 2020 [26]	R	Japan	01/2018-02/2020	28	74 (70–82)^b^	19/9	UC	II	Pembrolizumab	6 (3–18)^b^	SMI	OS, PFS
Shimizu et al. 2020 [27]	R	Japan	12/2017-08/2019	27	73 (52–82)^a^	23/4	UC	II	Pembrolizumab	7 (1–20)^a^	PMI	OS, PFS
Takei et al. 2024 [28]	R	Japan	2019–2023	60	71 (63–75)	46/14	RCC	I/II	Ipilimumab+Nivolumab	15 (1–52)^a^	SMI, VAI, SAI	OS, PFS

^a^medians with ranges; ^b^median and interquartile range; ^c^medians; ^d^<65/≥65; ^e^<75/≥75; R, retrospective study; UC, urothelial carcinoma; RCC, renal cell carcinoma; SMI, skeletal muscle index; PMI, psoas muscle index; VAI, visceral adiposity index; SAI, subcutaneous adiposity index; IAI, inter-muscular adiposity index; OS, overall survival; PFS, progression-free survival; ICI, immune checkpoint inhibitor.

### Association between SMI and the outcomes of UM patients with ICI therapy

**Fig. 2:**
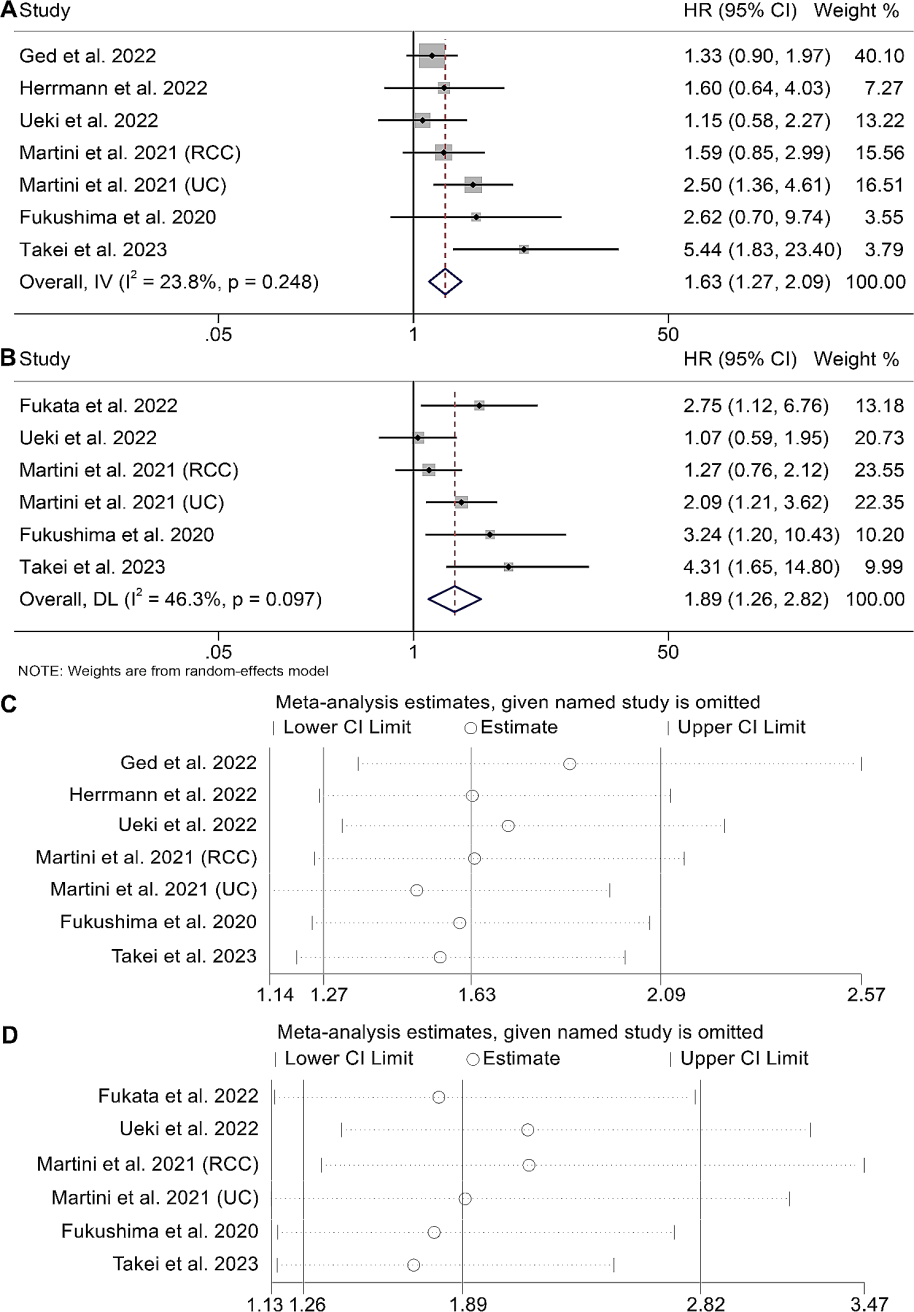
Forest plots of the relationship between SMI and overall survival in all included studies (**A**). Forest plots of the relationship between SMI and progression-free survival in all included studies (**B**). Sensitivity analysis of the association between SMI and overall survival (**C**) and progression-free survival (**D**). HR, hazard ratio; CL, confidence interval

Our analysis comprised seven studies, with a collective sample size of 628 patients, exploring the influence of SMI on UM patients’ outcomes following ICI treatment. The findings demonstrated that low SMI patients had a significantly worse OS than those with high SMI (HR: 1.63, 95% CI: 1.27–2.09, *p* < 0.001, Fig. 2A). No substantial heterogeneity was observed across the studies, so we adopted a fixed-effects model (I^2^ = 23.8%, *p* = 0.248). Additionally, we evaluated the correlation between SMI and PFS. The Cochran Q test and I^2^ statistics indicated no substantial heterogeneity (*p* = 0.097, I^2^ = 46.3%), and we employed a random-effect model. The results of our analysis revealed that UM patients with low SMI had a 89% higher risk of disease progression compared to those with higher SMI (Fig. 2B, HR: 1.89, 95% CI: 1.26–2.82, *p* = 0.002).

We performed evaluations to detect publication bias in the correlation between SMI and OS and PFS in the meta-analysis. No evidence of publication bias in either OS (Begg’s test: *p* = 0.230, Egger’s test: *p* = 0.122) or PFS (Begg’s test: *p* = 0.060, Egger’s test: *p* = 0.063) was shown. To further evaluate the robustness, a sensitivity analysis was conducted, and the combined HR for OS remained statistically significant and stable in our findings. The range of HRs varied from 1.50 (95% CI: 1.14–1.97) upon exclusion of the study conducted by Martini et al. (UC) to 1.87 (95% CI: 1.36–2.25) after excluding the study by Ged et al., as depicted in Fig. 2C. The pooled HR for PFS was also not substantially altered in the sensitivity analysis (Fig. 2D).

**Fig. 3:**
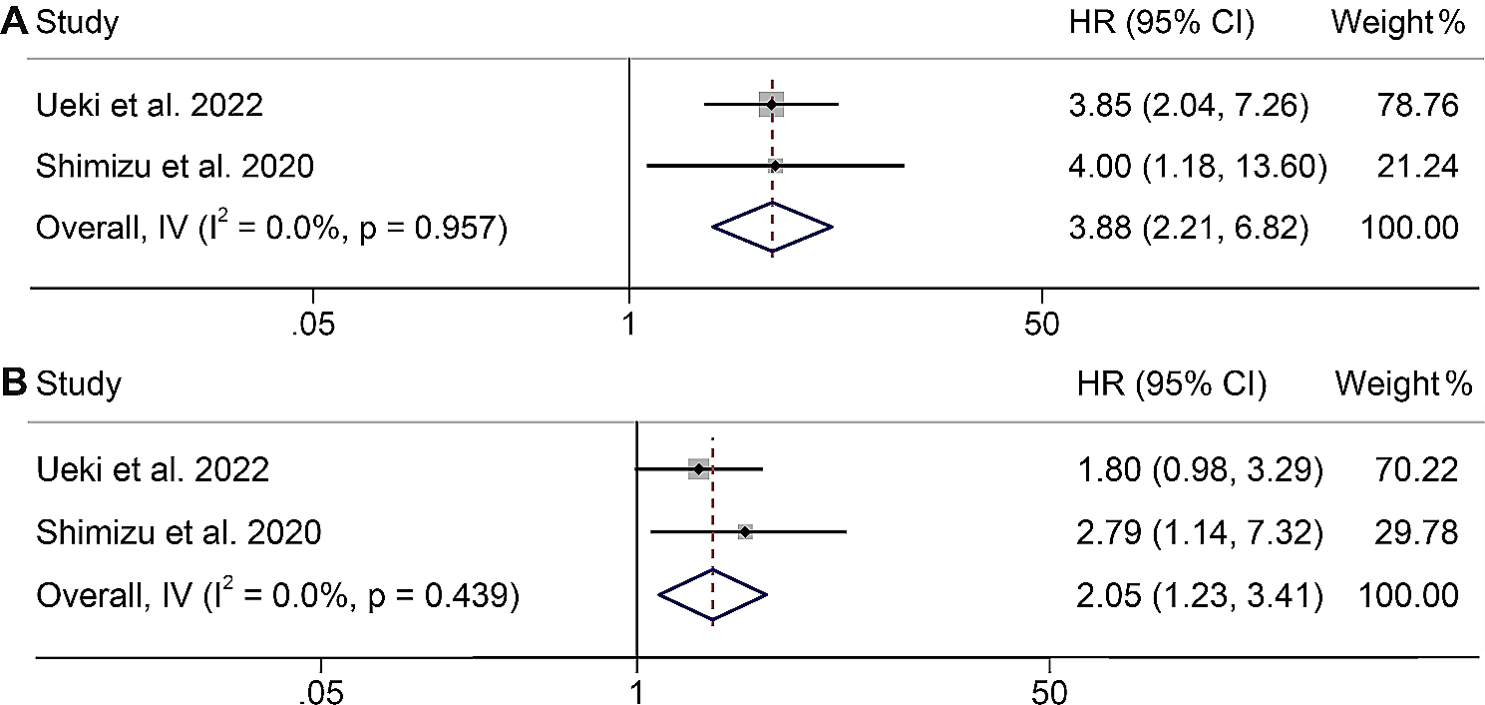
Forest plots of the relationship between PMI and overall survival in all included studies (**A**). Forest plots of the relationship between PMI and progression-free survival in all included studies (**B**). HR, hazard ratio; CL, confidence interval

### Association between PMI and the outcomes of UM patients with ICI therapy

We incorporated two studies to investigate the impact of psoas muscle index (PMI) on patients with UM undergoing ICI therapy. Our findings revealed that individuals with low PMI exhibited a poorer OS when contrasted with those with high PMI (HR: 3.88, 95% CI: 2.21–6.82, *p* < 0.001, Fig. 3A). There was no significant heterogeneity, as evidenced by the Cochran Q test and I^2^ statistics (*p* = 0.957, I^2^ = 0.0%), leading to the utilization of a fixed-effects model. Furthermore, we assessed the correlation between PMI and PFS in these patients, revealing that individuals with low PMI had a 105% elevated risk of disease progression compared to those with high PMI (HR: 2.05, 95% CI: 1.23–3.41, *p* = 0.006, Fig. 3B). A fixed-effects model was employed due to the absence of substantial heterogeneity among the included studies (I^2^ = 38.0%, *p* = 0.168).

### Association between sarcopenia and the outcomes of UM patients with ICI therapy

**Fig. 4:**
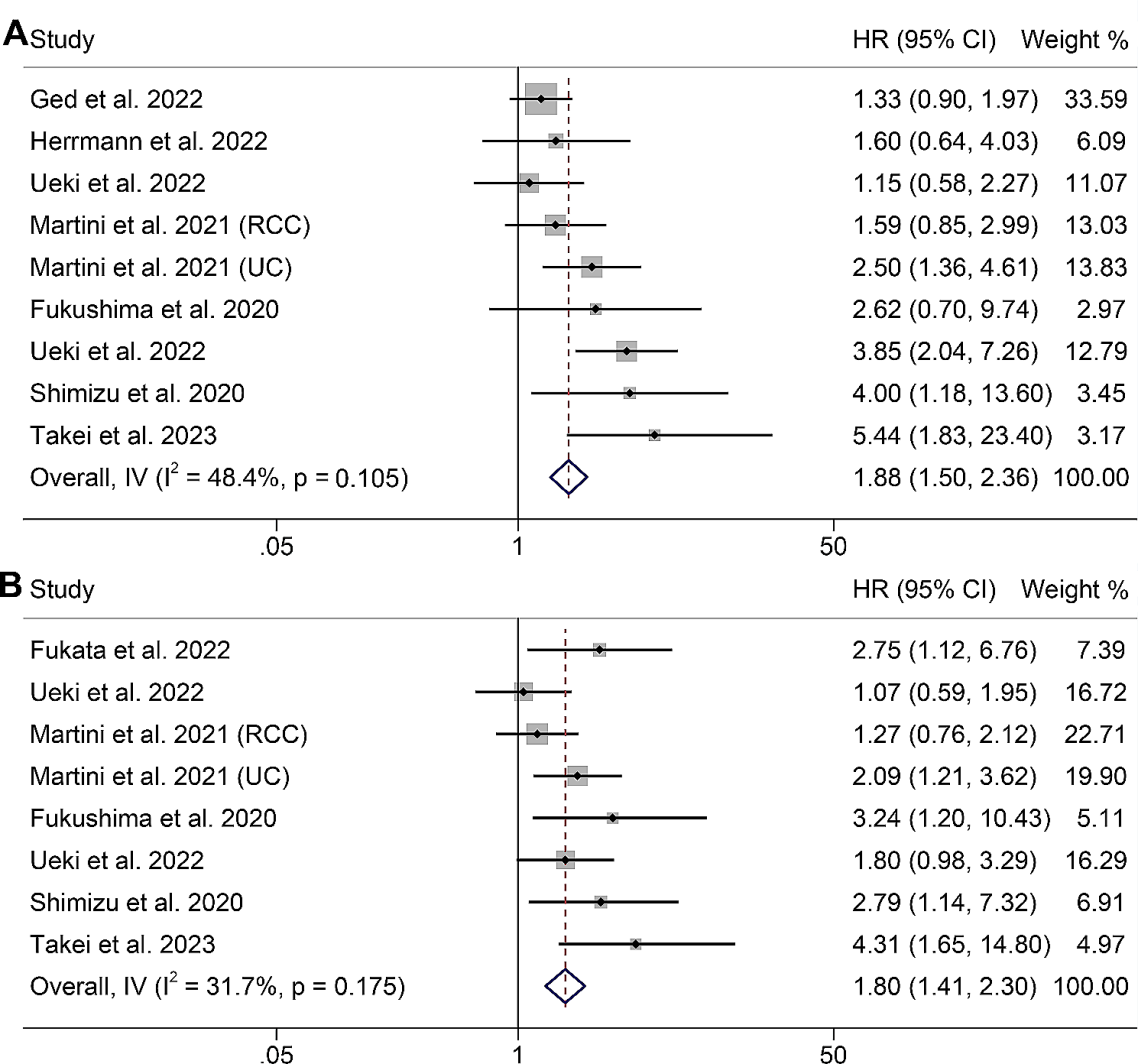
Forest plots of the relationship between sarcopenia and overall survival in all included studies (**A**). Forest plots of the relationship between sarcopenia and progression-free survival in all included studies (**B**). HR, hazard ratio; CL, confidence interval

As previously stated, the assessment of sarcopenia in the studies employed the SMI in seven of them, while PMI was utilized in two. The objective was to investigate the impact of sarcopenia on patients with UM undergoing immunotherapy with ICIs. Our analysis revealed that UM patients with sarcopenia exhibited markedly inferior OS in comparison to those without sarcopenia (HR: 1.88, 95% CI: 1.50–2.36, *p* < 0.001, as depicted in Fig. 4A). Since the Cochran Q test and I^2^ statistics indicated no significant heterogeneity among the studies (*p* = 0.105, I^2^ = 48.4%), we employed a fixed-effects model. Furthermore, we investigated the association between sarcopenia and PFS in these patients. Our analysis demonstrated that individuals with sarcopenia had a 80% elevated risk of disease progression compared to those without sarcopenia (HR: 1.80, 95% CI: 1.41–2.30, *p* < 0.001, Fig. 4B). The statistical assessments revealed no substantial heterogeneity among the studies (*p* = 0.175, I^2^ = 31.7%), enabling the adoption of a fixed-effects model. A connection between sarcopenia and Disease control rate (DCR) in UM patients was observed. No significant heterogeneity was included in the studies (I^2^ = 68.6%, *p* = 0.013), and a random-effects model was applied. We found that UM patients with sarcopenia had a lower DCR (OR: 0.43, 95% CI: 0.20–0.94, Figure S3) than those without sarcopenia.

Moreover, we conducted tests to assess potential publication bias in the combined analysis of the correlation between sarcopenia and both OS and PFS. The results indicated the absence of publication bias in OS (Begg’s test: *p* = 0.251, Egger’s test: *p* = 0.109), while there was evidence of publication bias in PFS (Begg’s test: *p* = 0.019, Egger’s test: *p* = 0.017). Henceforth, additional validation through the implementation of the trim and fill technique was utilized to rectify probable publication bias. Nevertheless, the aggregated results, which had demonstrated significance prior to employing the “trim and fill” strategy, retained their significance post-adjustment (HR: 1.61, 95% CI: 1.18–2.21, *p* = 0.003), implying negligible influence of this publication bias on the pooled estimations.

**Fig. 5:**
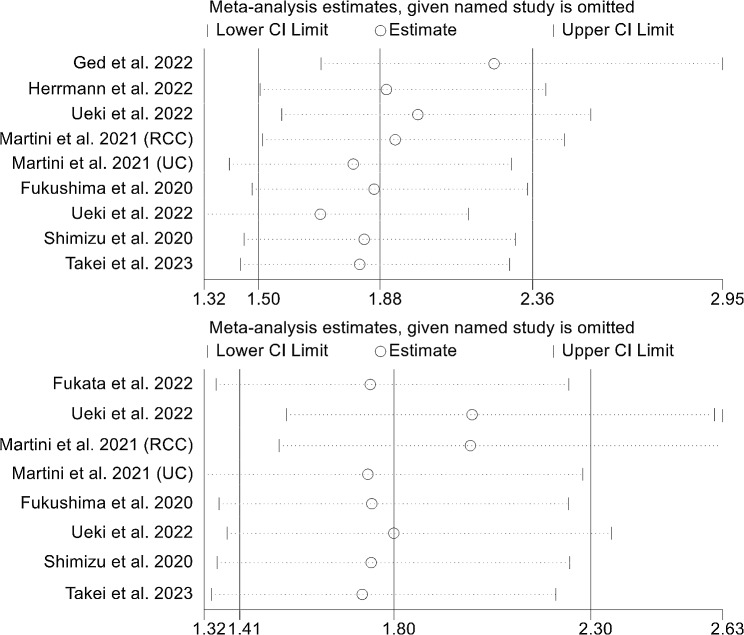
Sensitivity analysis of the association between sarcopenia and overall survival (**A**) and progression-free survival (**B**). CL, confidence interval

Additionally, the leave-one-out method was employed in a sensitivity analysis to investigate the potential influence of each study on the pooled results. The findings revealed that the HR for OS remained stable and reliable, ranging from 1.69 (95% CI: 1.32–2.15, after excluding Ueki et al.) to 2.23 (95% CI: 1.69–2.95, after excluding Ged et al., Fig. 5A). Similarly, the HR for PFS did not substantially vary during the sensitivity analysis (Fig. 5B). Based on these results, we can confidently conclude that our findings are robust and dependable.

### Association between VAI and the outcomes of UM patients with ICI therapy

**Fig. 6:**
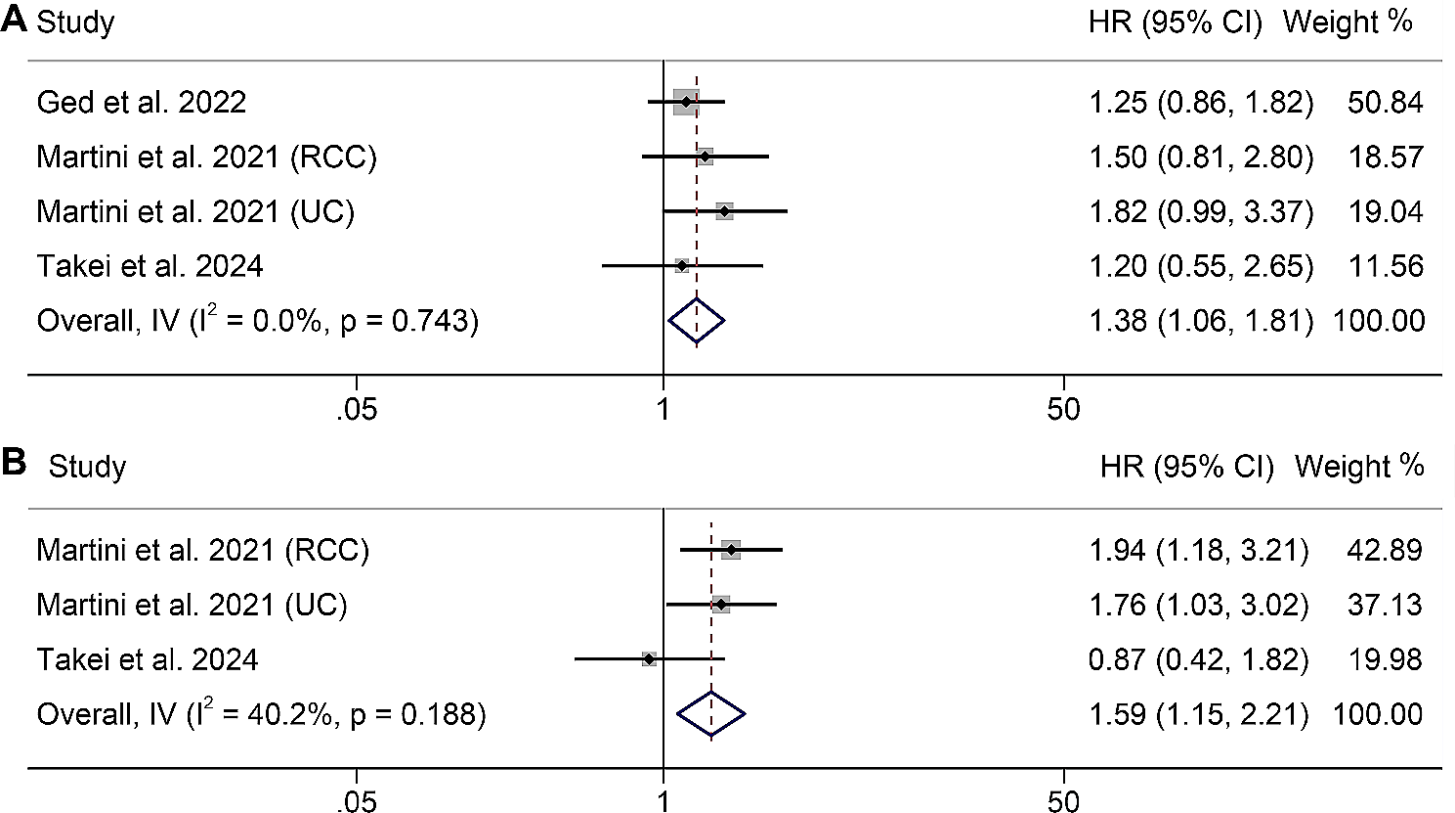
Forest plots of the relationship between VAI and overall survival in all included studies (**A**). Forest plots of the relationship between VAI and progression-free survival in all included studies (**B**). HR, hazard ratio; CL, confidence interval

In our study, the impact of VAI on UM patients undergoing ICI therapy was examined in four studies. Our analysis revealed that UM patients receiving ICI therapy with low VAI had a significantly poorer OS compared to those with high VAI (HR: 1.38, 95% CI: 1.06–1.81, *p* = 0.018, Fig. 6A). No heterogeneity was observed (I^2^ = 0, *p* = 0.743), with the use of a fixed-effects model. Additionally, we investigated the correlation between VAI and PFS in UM patients undergoing ICI therapy. No significant heterogeneity was observed among the studies (I^2^ = 40.2%, *p* = 0.188), so we used a fixed-effects model. Our meta-analysis demonstrated that patients with low VAI had an 59% greater risk of progression than those with high VAI (HR:1.59, 95% CI: 1.15–2.21, *p* = 0.005, Fig. 6B).

### Association between SAI and the outcomes of UM patients with ICI therapy

We examined five studies to assess the influence of SAI on the outcomes of UM patients undergoing treatment with ICIs. Our analysis revealed that patients with low SAI had significantly shorter OS than those with high SAI (HR: 1.37, 95% CI: 1.05–1.77, *p* = 0.018, Figure S1A). We observed no significant heterogeneity among the studies, allowing us to use a fixed-effects model for the analysis. However, low SAI did not predict the efficacy of ICIs for PFS (HR: 1.10, 95% CI: 0.65–1.87, *p* = 0.712, Figure S1B). Thus, while low SAI was associated with worse OS in UM patients, it was not a reliable predictor of ICI efficacy.

### Association between IAI and the outcomes of UM patients with ICI therapy

We performed a meta-analysis of two studies to investigate the association between IAI and patient outcomes in UM undergoing treatment with ICIs. Due to significant heterogeneity, a random-effects model was utilized for the analysis of both OS and PFS (OS: I^2^ = 85.6%, *p* = 0.008, Figure S2A; PFS: I^2^ = 88.3%, *p* = 0.003, Figure S2B). The results of our analysis showed no significant association between IAI and either OS (HR: 0.865, 95% CI: 0.271–2.762, *p* = 0.806, Figure S2A) or PFS (HR: 0.842, 95% CI: 0.283–2.504, *p* = 0.757, Figure S2B).

## Discussion

The administration of ICIs for UM treatment has grown in popularity, and researchers have been striving to identify factors that affect their effectiveness. The influence of baseline body composition on ICI response in UM patients is still a topic of controversy. Our work aims to investigate the association between baseline body composition and ICI efficacy in UM patients by synthesizing all available evidence. Our analysis shows that baseline body composition, including decreased PMI, SMI, sarcopenia, and VAI, were significantly linked with OS and PFS in ICI-treated UM patients. SAI exhibited a significant association with OS, while its correlation with PFS did not reach statistical significance. Conversely, no significant correlation was found between IAI and OS or PFS. Our publication bias and sensitivity analyses lend credence to our findings. Therefore, our study is crucial in providing novel biomarkers for patients undergoing ICI treatment.

The use of axial computed tomography (CT) software for determining muscle mass and density has been widely accepted as an objective and reproducible method for diagnosing sarcopenia. At present, there are two CT-based methods for quantifying skeletal muscle mass, which include SMI and PMI [29]. A meta-analysis has confirmed that sarcopenia is significantly associated with a poor prognosis in oncology [30]. Earlier research studies have indicated that sarcopenia is a crucial predictor of prognosis in individuals suffering from metastatic renal cell carcinoma (mRCC) [31]. Additionally, research studies have reported that PMI-based sarcopenia is also an important prognostic factor in RCC patients with nivolumab therapy [27]. Moreover, previous research has suggested that sarcopenia may be useful in predicting the response to PD-1 inhibitors [32]. For instance, Cortellini et al. found that a decreased SMI was associated with reduced survival in advanced cancer patients who were treated with PD-1/PD-L1 checkpoint inhibitors, including those with melanoma, RCC, and lung cancer [33]. Similarly, Takenaka covered that patients with sarcopenia had poorer survival and response rates to ICIs and that sarcopenia could predict the efficacy of various types of tumors [29]. These findings are consistent with our work. There is evidence that exercise and non-sarcopenic status can boost tumor immunity, including the production of natural killer cells [34]. Therefore, preserving skeletal muscle mass might enhance the effectiveness of ICIs. As a result, therapeutic interventions such as nutritional support, exercise, and medication may play a crucial role in improving sarcopenia and maximizing the benefits of ICIs.

Sarcopenia may affect the efficacy of ICIs in various ways. Chronic tumor-related inflammation, which can cause sarcopenia, may contribute to tumor cell immune evasion by inducing T-cell exhaustion [35]. Recently, skeletal muscle has been identified as an endocrine organ that secretes cytokines known as myokines [36]. Okumura et al. have suggested that decreased muscle mass can result in reduced production of myokines, which may negatively impact immunity [37]. Skeletal muscle produces various myokines, including interleukin (IL)-6, IL-8, and IL-15. It has been shown by Waldmann that IL-15 can increase the proportion of circulating natural killer cells and CD8^+^ T cells [38]. Therefore, sarcopenia-induced alterations in myokine levels could potentially influence the effectiveness of ICI therapy, suggesting the prognostic significance of sarcopenia in immune-based interventions.

Furthermore, studies have shown that adipose tissue in obese mice has fewer regulatory T cells (Tregs) and effector T cells, along with an elevated CD8^+^/CD4^+^ ratio [39]. In lean mice, Tregs act as inhibitors of the inflammatory process in adipose tissue. In contrast, the number of Tregs is significantly reduced in the adipose tissue of obese mice. Experimental evidence suggests that overweight individuals with pre-existing malignancies may exhibit increased susceptibility to checkpoint inhibition due to a pro-inflammatory state. This state is characterized by elevated Th1 responses, macrophage polarization towards the M1 pro-inflammatory phenotype, and a decreased Treg population in adipose tissue. This indicates that the adipose tissue in overweight individuals creates an inflammatory microenvironment that may influence the effectiveness of ICIs and impact the immune response against malignancies [40]. However, this hypothesis needs to be validated with more clinical data and further experiments. Therefore, it is crucial to consider the potential impact of body composition on ICI efficacy in clinical practice. Further research is needed to determine whether body composition could enhance the therapeutic benefits of ICI immunotherapy and to elucidate the underlying mechanisms.

This study has several strengths. Firstly, it employed a meta-analysis approach that analyzed a significant number of patients. The inclusion of small-scale retrospective studies enabled us to gather more reliable predictions on the relationship between body composition and clinical outcomes in UM patients undergoing ICI therapy. Secondly, the study utilized extensive criteria for body composition measurements, which allowed us to determine the most suitable method for predicting ICI effectiveness.

However, the article also has some limitations. Firstly, as a meta-analysis, this study depended on previously published literature, which may have limited the availability of data for conducting subgroup analyses based on various types and dosages of ICIs. Secondly, all the studies included in the analysis were retrospective in nature, which may have inherent limitations such as selection and reporting bias. Finally, most of the included studies were calculated based on univariate analysis, without some potential confounding factors (such as exercise status, other comorbidities, etc.), which may have some influence on the results. Hence, further extensive prospective studies are required to comprehensively investigate the correlation between body composition and clinical outcomes in UM patients receiving ICI therapy.

## Conclusions

The results of our meta-analysis indicate an effective association between changes in body composition and decreased clinical benefit in UM patients.

### Electronic supplementary material

Below is the link to the electronic supplementary material.

Supplementary Material 1

Supplementary Material 2

Supplementary Material 3

Supplementary Material 4

Supplementary Material 5

Supplementary Material 6

Supplementary Material 7

## Data Availability

The data that support the findings of this study are available from the corresponding author upon reasonable request.
