# Correction: Impact of baseline body composition on prognostic outcomes in urological malignancies treated with immunotherapy: a pooled analysis of 10 retrospective studies

**DOI:** 10.1186/s12885-024-12638-3

**Published:** 2024-07-16

**Authors:** Wangbin Ma, Qiao Shi, Lilong Zhang, Zhendong Qiu, Tianrui Kuang, Kailiang Zhao, Weixing Wang

**Affiliations:** 1 Department of General Surgery, Renmin Hospital of Wuhan University, Wuhan, China; 2 Hubei Key Laboratory of Digestive System Disease, Wuhan, China; 3 Laboratory of General Surgery, Renmin Hospital of Wuhan University, Wuhan, China

**Correction:** **BMC Cancer 24, 830 (2024)**

**https://doi.org/10.1186/s12885-024-12579-x**

Following publication of the original article [1], a typesetting error was reported. The initials of author Kailiang Zhao (ZK) were erroneously added to the author group. This has been removed in this correction article.

Further to this, the authors reported an error in Table 1, whereby the last row in the table was omitted.

The incorrect Table 1:

**Table 1:** Main characteristics of the studies included

**Study**	**Study design**	**Study region**	**Study period**	**Sample size**	**Age**	**Male/female**	**Site**	**Treatment line**	**Treatment**	**Follow-up (months)**	**Body Composition Variables**	**Outcomes**
Aslan et al. 2022 [19]	R	Turkey	10/2010-10/2021	52	30/22^d^	38/14	RCC	II	Nivolumab	11.4 (0.7–63)^a^	SAI	OS, PFS
Fukata et al. 2022 [20]	R	Japan	02/2018-03/2021,	44	70 (54–80)^a^	30/14	UC	II	Pembrolizumab	13.2(1–40.8)^a^	SMI	PFS
Ged et al. 2022 [21]	R	USA	07/2011-04/2020	205	63 (40–90)^a^	152/53	RCC	I/II	ICIs	31.2 (1-77.8)^a^	SMI, VAI, SAI	OS
Herrmann et al. 2022 [22]	R	France	2016–2020	46	66 (37–86)^a^	31/13	RCC	II	Nivolumab	16^c^	SMI	OS
Ueki et al. 2022 [23]	R	Japan	12/2016-10/2020	96	65/31^e^	71/25	RCC	II	Nivolumab	9.7 (0.3–48.4)^a^	PMI, SMI	OS, PFS
Martini et al. 2021(RCC) [24]	R	USA	2015–2020	79	61.0^c^	58/21	RCC	I/II	ICIs	-	SMI, VAI, SAI, IAI	OS, PFS
Martini et al. 2021(UC) [25]	R	USA	2015–2020	70	69.5^c^	49/21	UC	I/II	ICIs	20.1^c^	SMI, VAI, SAI, IFI	OS, PFS
Fukushima et al. 2020 [26]	R	Japan	01/2018-02/2020	28	74 (70–82)^b^	19/9	UC	II	Pembrolizumab	6 (3–18)^b^	SMI	OS, PFS
Shimizu et al. 2020 [27]	R	Japan	12/2017-08/2019	27	73 (52–82)^a^	23/4	UC	II	Pembrolizumab	7 (1–20)^a^	PMI	OS, PFS

^a^medians with ranges; ^b^median and interquartile range; ^c^medians; ^d^ < 65/ ≥ 65; ^e^ < 75/ ≥ 75; R, retrospective study; UC, urothelial carcinoma; RCC, renal cell carcinoma; SMI, skeletal muscle index; PMI, psoas muscle index; VAI, visceral adiposity index; SAI, subcutaneous adiposity index; IAI, inter-muscular adiposity index; OS, overall survival; PFS, progression-free survival; ICI, immune checkpoint inhibitor

The corrected Table 1:

**Table 1:** Main characteristics of the studies included

**Study**	**Study design**	**Study region**	**Study period**	**Sample size**	**Age**	**Male/female**	**Site**	**Treatment line**	**Treatment**	**Follow-up (months)**	**Body Composition Variables**	**Outcomes**
Aslan et al. 2022 [19]	R	Turkey	10/2010-10/2021	52	30/22^d^	38/14	RCC	II	Nivolumab	11.4 (0.7–63)^a^	SAI	OS, PFS
Fukata et al. 2022 [20]	R	Japan	02/2018-03/2021,	44	70 (54–80)^a^	30/14	UC	II	Pembrolizumab	13.2(1–40.8)^a^	SMI	PFS
Ged et al. 2022 [21]	R	USA	07/2011-04/2020	205	63 (40–90)^a^	152/53	RCC	I/II	ICIs	31.2 (1-77.8)^a^	SMI, VAI, SAI	OS
Herrmann et al. 2022 [22]	R	France	2016–2020	46	66 (37–86)^a^	31/13	RCC	II	Nivolumab	16^c^	SMI	OS
Ueki et al. 2022 [23]	R	Japan	12/2016-10/2020	96	65/31^e^	71/25	RCC	II	Nivolumab	9.7 (0.3–48.4)^a^	PMI, SMI	OS, PFS
Martini et al. 2021(RCC) [24]	R	USA	2015–2020	79	61.0^c^	58/21	RCC	I/II	ICIs	-	SMI, VAI, SAI, IAI	OS, PFS
Martini et al. 2021(UC) [25]	R	USA	2015–2020	70	69.5^c^	49/21	UC	I/II	ICIs	20.1^c^	SMI, VAI, SAI, IFI	OS, PFS
Fukushima et al. 2020 [26]	R	Japan	01/2018-02/2020	28	74 (70–82)^b^	19/9	UC	II	Pembrolizumab	6 (3–18)^b^	SMI	OS, PFS
Shimizu et al. 2020 [27]	R	Japan	12/2017-08/2019	27	73 (52–82)^a^	23/4	UC	II	Pembrolizumab	7 (1–20)^a^	PMI	OS, PFS
Takei et al. 2024 [28]	R	Japan	2019-2023	60	71 (63-75)	46/14	RCC	I/II	Ipilimumab+Nivolumab	15(1-52) ^a^	SMI, VAI, SAI	OS, PFS

^a^medians with ranges; ^b^median and interquartile range; ^c^medians; ^d^ < 65/ ≥ 65; ^e^ < 75/ ≥ 75; R, retrospective study; UC, urothelial carcinoma; RCC, renal cell carcinoma; SMI, skeletal muscle index; PMI, psoas muscle index; VAI, visceral adiposity index; SAI, subcutaneous adiposity index; IAI, inter-muscular adiposity index; OS, overall survival; PFS, progression-free survival; ICI, immune checkpoint inhibitor

The original article [1] has been corrected.
