# Supplementary figures and images for: Impact of baseline body composition on prognostic outcomes in urological malignancies treated with immunotherapy: a pooled analysis of 10 retrospective studies
Source: BMC Cancer. 2024 Jul 11;24:830. doi: 10.1186/s12885-024-12579-x (PMC11241896; doi:10.1186/s12885-024-12579-x)

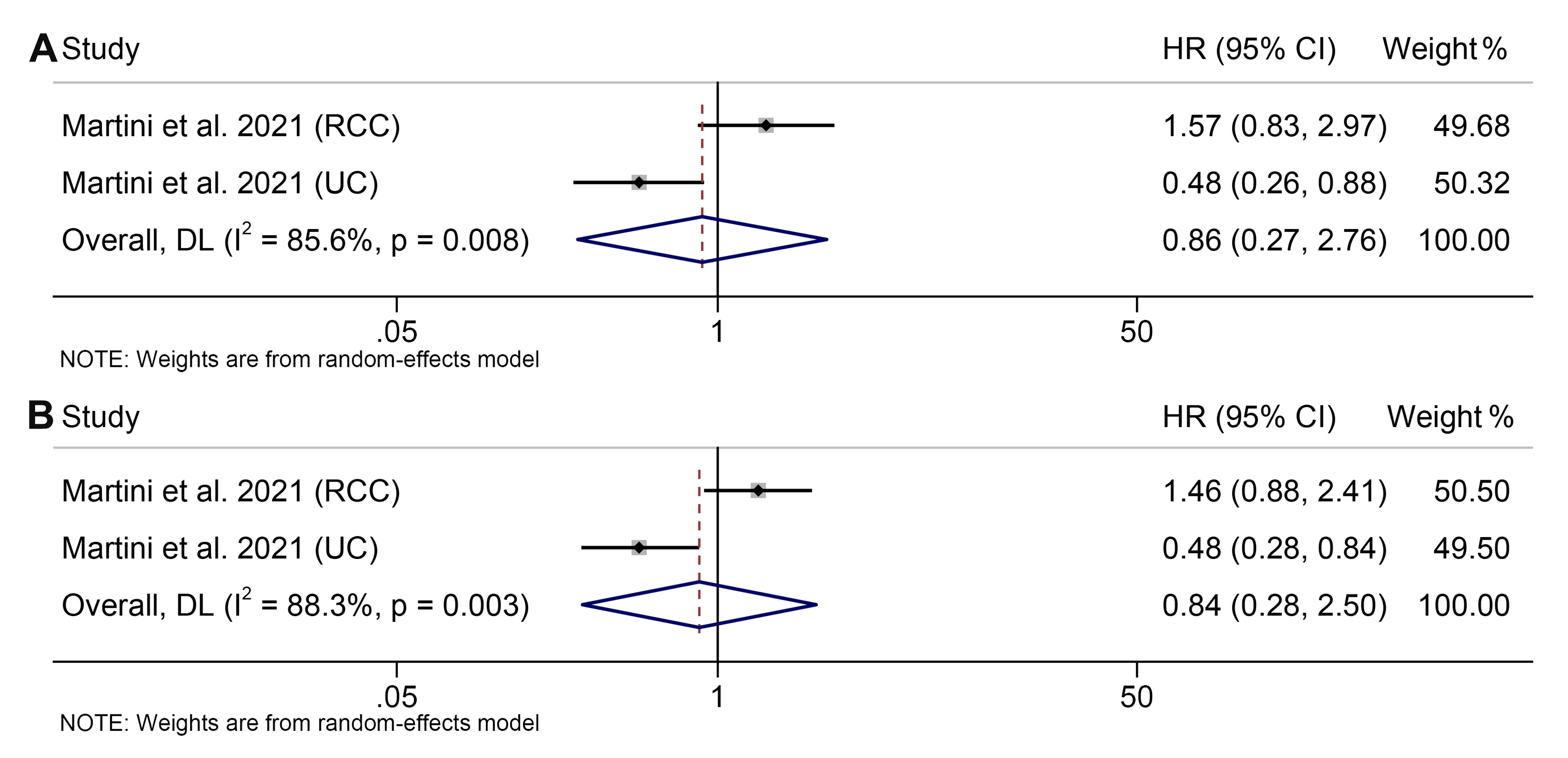

Supplement: Supplementary file 2 — Supplementary Material 2 [file 12885_2024_12579_MOESM2_ESM.png]

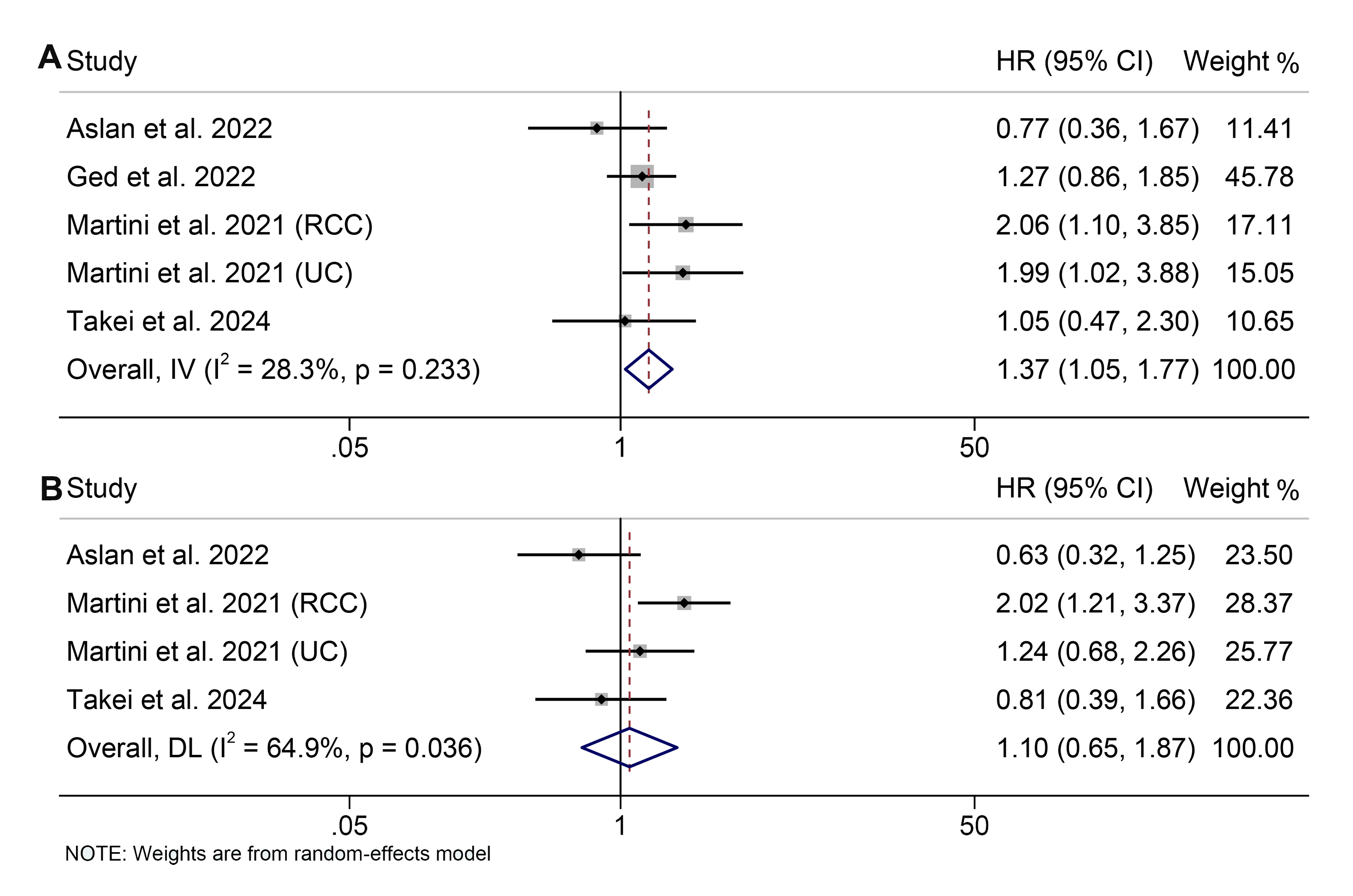

Supplement: Supplementary file 3 — Supplementary Material 3 [file 12885_2024_12579_MOESM3_ESM.png]

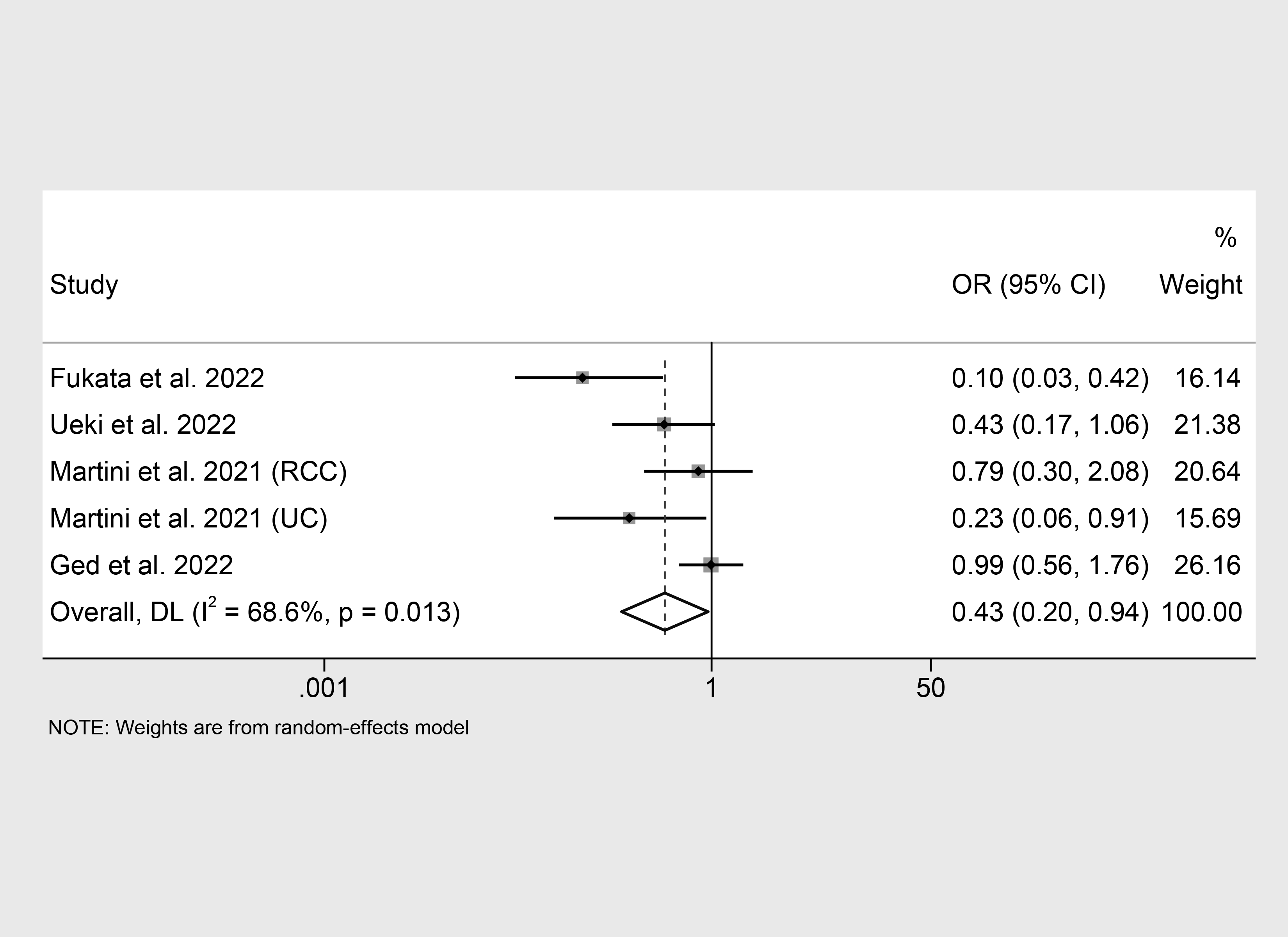

Supplement: Supplementary file 4 — Supplementary Material 4 [file 12885_2024_12579_MOESM4_ESM.png]
