# Supplementary material for: Impact of baseline body composition on prognostic outcomes in urological malignancies treated with immunotherapy: a pooled analysis of 10 retrospective studies
Source: BMC Cancer. 2024 Jul 11;24:830. doi: 10.1186/s12885-024-12579-x (PMC11241896; doi:10.1186/s12885-024-12579-x)
Supplement: Supplementary file 7 — Supplementary Material 7 [file 12885_2024_12579_MOESM7_ESM.docx]

Table S1 Detailed search strategy.

Table S2 The definitions of SMI, PMI, SAI, VAI, IAI, and sarcopenia in the included studies.

Table S3 PRISMA_2020_checklist.

Figure S1 Forest plots of the relationship between SAI and overall survival in all included studies (A). Forest plots of the relationship between SAI and progression-free survival in all included studies (B). HR, hazard ratio; CL, confidence interval.

Figure S2 Forest plots of the relationship between IAI and overall survival in all included studies (A). Forest plots of the relationship between IAI and progression-free survival in all included studies (B). HR, hazard ratio; CL, confidence interval.

Figure S3 Forest plots of the relationship between sarcopenia and Disease control rate in all included studies.
